# Charles Mercier's Life Reflected in His Work

**Published:** 1919-09-13

**Authors:** 


### Charles Mercier's Life Reflected in His Work.

The adventurous energy which led Dr. Mercier to try the varied occupations of his youth and to succeed at last in medicine, is reflected in the varied subjects which occupied his pen, particularly in the last twenty years of his life. He learnt first in the school of experience, and consequently he made experience rather than reflection the test by which every problem was to be tackled. The virtue of his method consists in the trenchancy of the arguments which it supplied, in the absence of vague conclusions, and in the freshness of observation brought to bear upon the matter in hand. But the method, like any other, has its defects; and the defects are to be limited by the range of what is, after all, but the observation of one man, however able; to leave his prejudices uncorrected by that restraint which the study of literature engenders, to be cocksure rather than considerate, and to undervalue tradition and the consensus which in the course of ages has favoured one side rather than another in the old eternal controversies. Such an admission enables us to understand why Dr. Mercier's books, always distinguished and arresting, were yet very unequal, and explains why, when his subject was a question of fact, he was a safer guide than when it was a question of theory.

Characteristically enough, he was fond of writing text-books, and as a writer of text-books, when on subjects of which he had a firsthand acquaintance, he remains inimitable. Of the text-books, such as his "Text-book of Insanity," or the more technical "Lunacy Law for Medical Men," the first is a classic, and the second might have become so had its subject not been the caprice of legislation. Unlike most writers of text-books, Dr. Mercier had a literary gift of a very high order. The pen was his by right of birth. The right words came by instinct. His pen never fumbled, and the subject of insanity was one which he had thoroughly explored. His book, therefore, was brilliantly written, fresh from the mint of experience, a little masterpiece of its kind. It had the hall-mark of a true style, the note of authority. So delightful was it to read, that students were tempted to wonder whether it could really help them in their examinations. It seemed too interesting, too alive, too little of a cram. But they found that it helped them past belief, because it *contained* the subject, and enabled them to stand at its centre, regarded from which the facts, which are usually crammed, fell, as it were, by magic into their proper places in their memory. Consequently, his "Text Book of Insanity," had something of a general welcome, and this welcome encouraged its author to write learned books not only for students but for the public.

So his pen became engaged in the task of enlightening his fellow-countrymen over an ever-widening field. He gave them a book on "Criminal Responsibility," another on "Diseases of Conduct," and came to be in request by the publishers of popular hand-books. The practical value of his work can be illustrated by his attack upon the test of insanity then, and still to a large extent, in vogue, in courts of law. He declared that only confused judgment and unsatisfactory verdicts attended the practice of defining insanity to be a mental disease. He said that what the courts were, or ought to be, concerned with was not any mental condition at all. They were, or ought to be, concerned with people's conduct. Consequently people's acts, and not their mental state, was the issue to be decided. A person might act in a way so contrary to decency or social order, that the repetition of such an action was clearly a good reason for placing its perpetrator in an asylum, whether or no the state of his mind, judged by itself, seemed or did not seem unsound. There could be, and generally was, much dispute about the latter. There rarely was any dispute whether it was desirable to allow the person to be, or not to be, controlled. The law was concerned with men's conduct, not with their mentality, and should lock them up or leave them free according to their behaviour. That, crudely stated, was his view; and the simplicity of the test, the concreteness of the issue, has recommended itself increasingly. Here, again, the issue was practical, and, like all practical matters, limited. Here Dr. Mercier was again at his best. His earlier book on "Criminal Responsibility" received its complement in "Diseases of Conduct." Both cleared the air, and, even in points where they were criticised, did not make confusion but cleared it away. For a negative merit of Dr. Mercier, and a very valuable one, was this: that if he did make a mistake it was sure to be a howler, and for that reason patent to everyone except himself.

His third literary period may be said to have begun by the publication of his "New Logic," which was issued after his unsuccessful candidature for a professorship of philosophy at Oxford. It consisted of an attack upon the Aristotelian Logic, and an attempt to replace that system by one on what he supposed to be fresh lines. Now here Dr. Mercier was concerned with a theoretic subject, the correct method of reasoning, and consequently he was not so sure as he thought he was of his ground. Attacks upon the syllogism are not new. Formal logic has been a historic target, though it is true that formal logic is still the system which is taught in the schools. That, perhaps, was enough to justify Dr. Mercier's onslaught.

After this effort, he became the chief writer of a new publishing house, and wrote for it a series of hand-books on "A Rational System of Education," "Spiritualism," an ironical criticism of Sir Oliver Lodge's "Raymond," and the like. Education is a dangerous subject. Dr. Mercier's view is, we believe, mistaken; but one can learn more from his mistakes than from the correctness of most writers on Education. He attacks the classical education on the ground that the claims advanced on behalf of classical subjects are un-

**Charles Arthur Mercier, M.D.—(*continued*).**

true. He says that our laws are not derived from Rome, nor our thought from Greece, and that the proper study of Englishmen is English. The last statement is true, as it stands; but "What do they know of England who only England know?" And, in truth, English cannot be mastered by Englishmen (since his own literary faculty is a rare gift) except by the study of the languages whence most of its words have been derived. To see English objectively, the unliterary man must get outside it, and that he can do best by the help of its root languages. The main argument for a classical education, however, is not this. It is rather that the higher education must begin at the beginning of European thought, which is Greece; and that we need to study a civilisation and a thought which are *unlike* our own, if we are to see our own in any sort of critical perspective. Because Greece is the only European civilisation, *not* Christian, of which we have a full record, therefore for us the value of its study is unique.

A reprint of a series of articles appeared in The Hospital on "Human Temperaments." Entertaining as they were, the classification of human temperaments into the "practical," the "artistic," the "artist's," the "jealous," and so forth, was too crude to be of much value. Human temperaments are more complex than that, and a jealous woman or practical man is something more subtle than a collection of the signs of jealousy or practicality. But he gives the stock signs of each type amusingly enough, and probably regarded their collection as something of a *jeu d'esprit*.

We have called his gifts rare. Good writers are rare; but good writers among medical men are very rare; and Dr. Mercier gave a literary standard to his profession for which that profession can never be sufficiently grateful.—O.B.

